# High-Intensity Focused Ultrasound Combined With Suction Curettage for the Treatment of Cesarean Scar Pregnancy

**DOI:** 10.1097/MD.0000000000000854

**Published:** 2015-05-08

**Authors:** Xiaogang Zhu, Xinliang Deng, Yajun Wan, Songshu Xiao, Jiping Huang, Lian Zhang, Min Xue

**Affiliations:** From the Department of Gynecology, Third Xiangya Hospital, Central South University, Changsha, Hunan Province (XZ, XD, YW, SX, MX); and State Key Laboratory of Ultrasound Engineering in Medicine, Chongqing Key Laboratory of Ultrasound in Medicine and Engineering, College of Biomedical Engineering, Chongqing Medical University, Chongqing, China (JH, LZ).

## Abstract

The aim of this study was to retrospectively evaluate the safety and feasibility of high-intensity focused ultrasound (HIFU) treatment combined with suction curettage under hysteroscopic guidance for cesarean scar pregnancy (CSP).

Fifty-three patients with definite CSP were treated with HIFU followed by suction curettage under hysteroscopic guidance. All the patients received 1 session of HIFU ablation under conscious sedation. Suction curettage under hysteroscopic guidance was performed at an average of 2.9 (range: 1–5) days after HIFU ablation. Blood flow of pregnancy tissue before and after HIFU, intraoperative blood loss in suction curettage and hysteroscopy procedure, time for β-human chorionic gonadotropin (β-hCG) to return to normal level, and time for normal menstruation recovery were recorded.

Immediately after HIFU treatment, color Doppler ultrasound showed that the fetal cardiac activity disappeared and the blood flow in the pregnancy tissue significantly decreased. All the patients underwent suction curettage under hysteroscopic guidance after the treatment of HIFU, the median volume of blood loss in the procedure was 20 mL (range: 10–400 mL). The average time for menstruation recovery was 35.1 ± 8.1 (range: 19–60) days. The average time needed for serum β-hCG to return to normal levels was 27.5 ± 6.4 (range: 12–40) days. The average hospital stay was 7.8 ± 1.5 (range: 5–11) days.

Based on our results, it appears that HIFU combined with suction curettage under hysteroscopic guidance is safe and effective in treating patients with CSP at gestational ages <8 weeks.

## INTRODUCTION

Cesarean scar pregnancy (CSP) is a special ectopic pregnancy and is mainly presented with the implantation of a gestational sac, fertilized egg, or embryo within a previous cesarean section scar. The incidence of CSP was reported from 1 in 2216 to 1 in 1800 pregnancies.^1^ Although this condition is rare, CSP may cause life-threatening complications such as uterine rupture and catastrophic hemorrhage. In China, the cesarean delivery rate almost reached 50%. This high incidence rate of cesarean section is likely to increase the prevalence of CSP.^2,3^ Unfortunately, to date, there has been no universal guideline on the management of this condition.^4^ Currently, surgical treatments and conservative treatment approaches are used in the management of CSP. Hysterectomy is a definite surgical treatment, but it is generally not ideal for these young patients.^5,6^ The main potential risk of conservative treatment for CSP is severe hemorrhage because curettage may rupture the uterine cesarean scar. Thus, several adjuvant treatments have been used to prevent massive bleeding. In addition, local or systemic administration of methotrexate (MTX) treatment plays a role in the management of CSP. It provides a non-invasive treatment option for patients who wish to preserve fertility with relatively low cost. However, this treatment is associated with a high failure rate of 57% and a high complication rate of 62.1%.^7,8^ Uterine artery embolization (UAE) is another option for patients with CSP. The success rate was higher than that of MTX, but the complication rate of UAE was still high (46.9%). Severe side effects such as infertility, infection, and ovarian dysfunction may occur after UAE.^9^

Over the last 10 years, ultrasound-guided high-intensity focused ultrasound (USgHIFU) has been used to treat solid tumors.^10–13^ In 2002, Wang et al^14^ reported their preliminary results of USgHIFU treatment for uterine fibroids. Many studies have shown that USgHIFU is safe and effective in treating uterine fibroids.^15,16^ Over the last few years, this non-invasive technique has also been used in treating adenomyosis.^17,18^ Therefore, we asked whether this non-invasive treatment could be used in the management of CSP. Recently, Huang et al reported their preliminary results for 4 patients with CSP treated using HIFU combined with dilation and curettage.^19^ In another study, 16 patients with CSP were treated with USgHIFU without using dilation and curettage. These preliminary results have shown that all patients’ serum beta human chorionic gonadotropin (β-hCG) levels returned to normal in 2 to 10 weeks, and the CSP mass disappeared at 2 to 14 weeks after 2 to 5 sessions of HIFU treatment.^20^ However, although their results have shown that USgHIFU seems to be feasible and effective in treating patients with CSP, the sample size was small and the protocol that they used was different. Herein, we retrospectively evaluated the results of 53 patients with CSP treated by HIFU and followed by suction curettage under hysteroscopic guidance in our institute to explore if and how USgHIFU can be safely used to treat CSP patients.

## MATERIALS AND METHODS

### Patients

**FIGURE 1:**
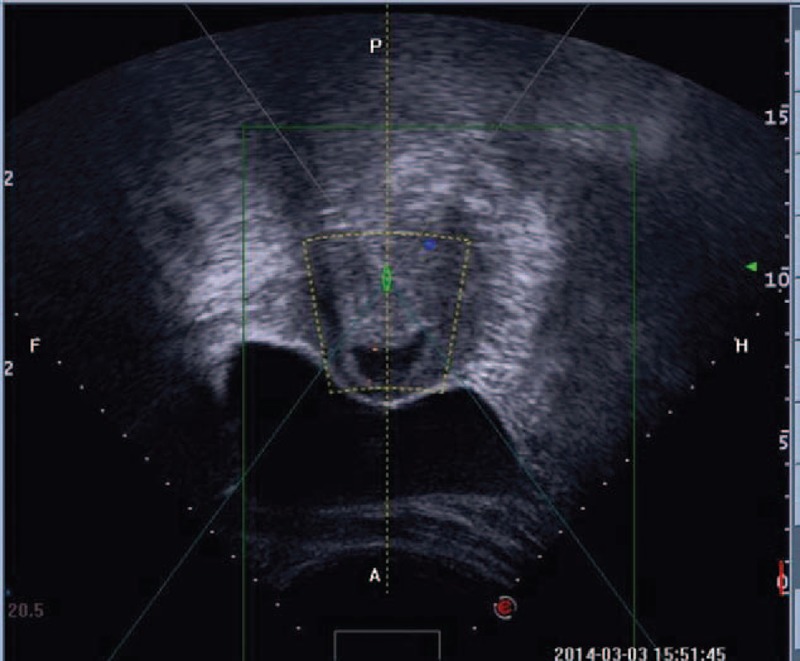
A gestational sac implanted in the previous cesarean scar with empty uterus cavity and cervical canal, and no myometrium was visible between the bladder and the sac.

**FIGURE 2:**
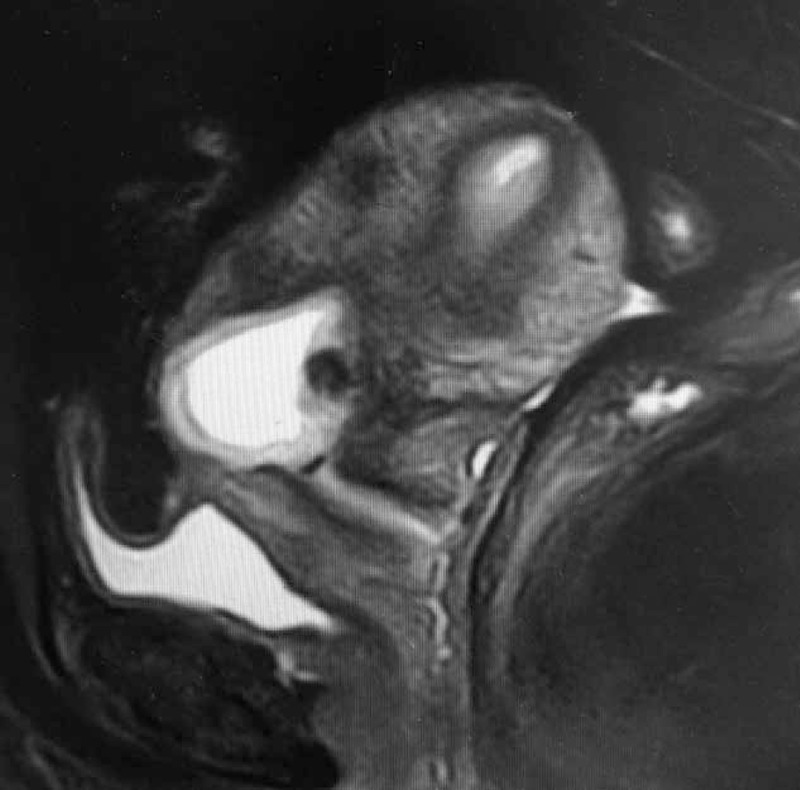
A gestational sac implanted in the previous cesarean scar (sagittal view of the magnetic resonance imaging).

Between January 2014 and September 2014, 53 patients with definite CSP were treated with HIFU followed by suction curettage under hysteroscopic guidance. The protocol was approved by the ethics committee and institutional review board of the institution. Written informed consent was obtained from every patient. Inclusion criteria for this study were: a history of cesarean section delivery; a history of amenorrhea and positive urine pregnancy test; ultrasound (Figure 1) and/or magnetic resonance imaging (Figure 2) indicating a CSP based on the diagnostic criteria, recommended by Godin et a^21^; gestational age <8 weeks. Exclusion criteria include: having pelvic inflammatory diseases; previous treatment for CSP before admission; other treatment for other diseases unrelated to CSP.

### Ultrasound-Guided HIFU Ablation

HIFU ablation was performed using the Haifu JC-200 focused ultrasound tumor therapeutic system (Chongqing Haifu Tech Co., Ltd., Chongqing, China). A transducer, with a 20-cm diameter and a focal length of 15 cm, produced the therapeutic energy required for treatment. An ultrasound imaging probe (My-Lab70, Esaote, Italy) is situated in the center of the transducer and allows real time sonographic monitoring during HIFU ablation. The transducer is located in a water reservoir filled with degassed water and its movement is controlled by a computer.

Before HIFU treatment, all patients were required to have specific bowel preparations. Every patient was suggested to ingest liquid food for 3 days, fast for 12 hours, and cleanse her bowel by enema. Every patient was requested to shave and degrease her anterior abdominal wall from the umbilicus to the level of upper margin of the pubic symphysis, and a urinary catheter was inserted to control the bladder volume.

Every patient only received 1 session of HIFU ablation. The procedure was performed under conscious sedation (fentanyl at 1 μg/kg, midazolam hydrochloride at 0.02 mg/kg, repeating administration of each on 40-min intervals if needed.). The patient was first positioned prone on the HIFU system with the anterior abdominal wall in contact with degassed water over the transducer in a sealed tank. During the procedure, real-time ultrasonography was used to determine the location of the target area of the gestational sac and to monitor the response to HIFU. Sagittal view of ultrasound scanning mode was selected, and the treatment plan was made by dividing the gestational sac into different slices with the thickness of 3 mm. The ablation procedure began from the innermost slice. The range of acoustic output power used was from 350 to 400 W. HIFU sonication terminated when the signal of the blood flow of pregnancy tissue disappeared or the gray scale change at the target tissue was observed on the color Doppler ultrasound. All patients were suggested to take oral prophylactic antibiotics for 3 days after HIFU treatment. Suction curettage under hysteroscopic guidance was performed at an average of 2.9 (range: 1–5) days after HIFU ablation.

### Suction Curettage Under Hysteroscopic Guidance

**FIGURE 3:**
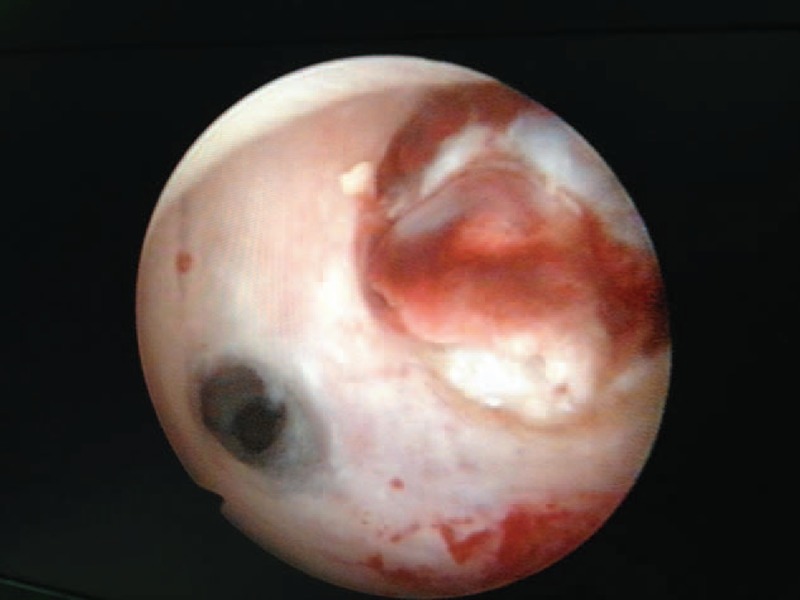
The pregnancy tissue located in the previous cesarean scar, with empty uterus cavity.

**FIGURE 4:**
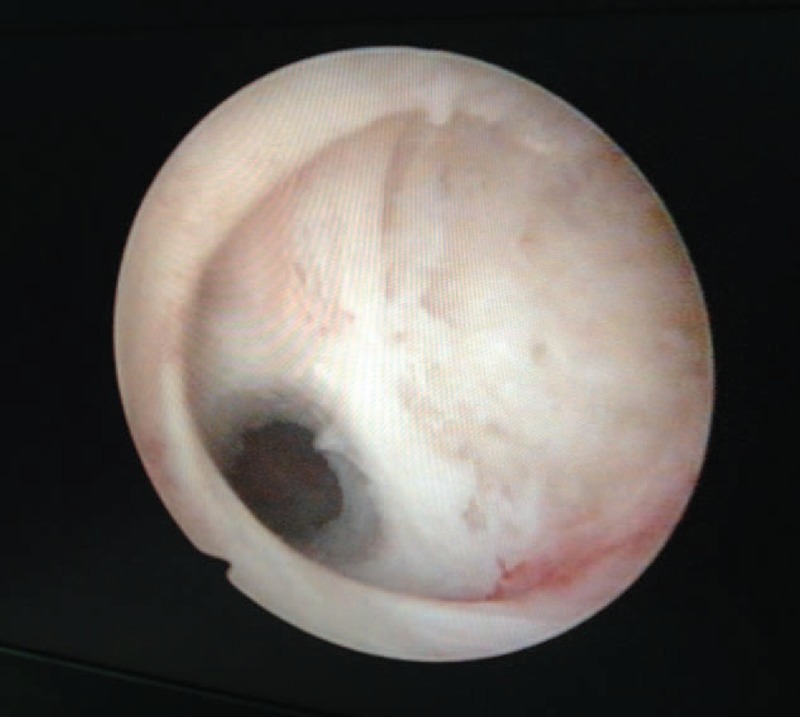
All the pregnancy tissues were removed from the previous cesarean scar.

Suction curettage under hysteroscopic guidance was performed under general anesthesia. The patients were placed in a dorsol lithotomy position. The depth of the uterus was measured before the procedure. A 30 degree diagnostic hysteroscope was used to visualize the uterine cavity and to locate the site of the pregnancy tissue in the myometrial defect (Figure 3). Normal saline was used as a distension medium. During the procedure, a 7- or 8-mm suction cannula was gently inserted into the uterine cavity, and the vacuum pressure was set at 0.04 MPa. The operator moved the cannula back and forth, rolling the cannula gently to detach the pregnancy tissues from the previous cesarean scar. Then, the hysteroscope was reinserted again to detect any retained pregnancy tissues from previous cesarean scars. If necessary, semiflexible forceps or a wire-loop electrode could be used to remove the retained pregnancy tissues (Figure 4). After the pregnancy tissues were removed, electrocoagulation was used to stop bleeding from the wound surface, and 10 mL of diluted oxytocin solution (0.24 unit/mL; 12 units of oxytocin diluted into 50 mL of normal saline) was injected into the cervical stroma at the 4 and 8 o’clock positions.

### Follow-Up Observation

All patients were discharged from hospital 2 days after suction curettage. Following the approved protocol, the serum β-hCG level was monitored weekly until it returned to normal. The patients were requested to come back to our department for a color Doppler ultrasound examination 1 month after taking suction curettage. The time of vaginal bleeding, abdominal pain, and the first return of the menstrual cycle were recorded.

### Statistical Methods

SPSS 17.0 software (SPSS, Inc, Chicago, IL) was used for statistical analysis. All data are presented as mean ± standard deviation (SD) or median.

## RESULTS

Fifty-three patients, between 22 and 44 years (31.8 ± 4.8), were enrolled in the study. Thirty-seven (69.8%) patients had 1 low-segment cesarean delivery, and 16 (30.2%) patients had 2 low-segment cesarean deliveries. Also, 5 (9.4%) patients were diagnosed with CSP for the second time and 48 (90.6%) patients were diagnosed with CSP for the first time. The median interval from the last cesarean section to CSP was 48 months (range: 4–218 months). The average gestational age was 47.7 ± 5.0 days (range: 37–56 days). Symptoms related to CSP included painless vaginal bleeding in 22 of 53 patients, vaginal bleeding with abdominal pain in 10 patients, and abdominal pain without vaginal bleeding in 1 patient. Twenty patients did not have subjective symptoms. Among these patients, the median serum β-hCG level before treatment was 36645 mIU/mL (range: 207.7–108257 mIU/mL), the median largest diameter of the sac/mass was 37 mm (range: 13–60 mm), and fetal cardiac activity was observed in 20 cases. The average thickness of the intervening myometrium between the gestation sac and the bladder was 3.7 ± 2.0 mm (range: 1–9 mm) (Table 1).

**TABLE 1:**
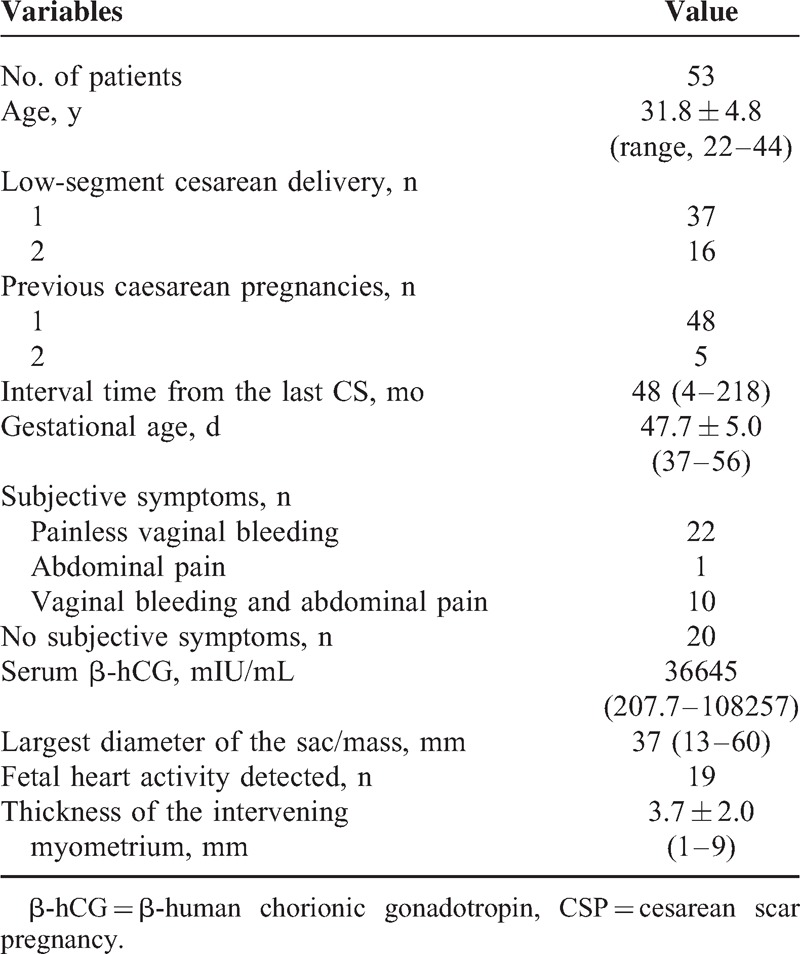
Demographic Characteristics of the Patients With CSP

### HIFU Ablation Evaluation

**FIGURE 5:**
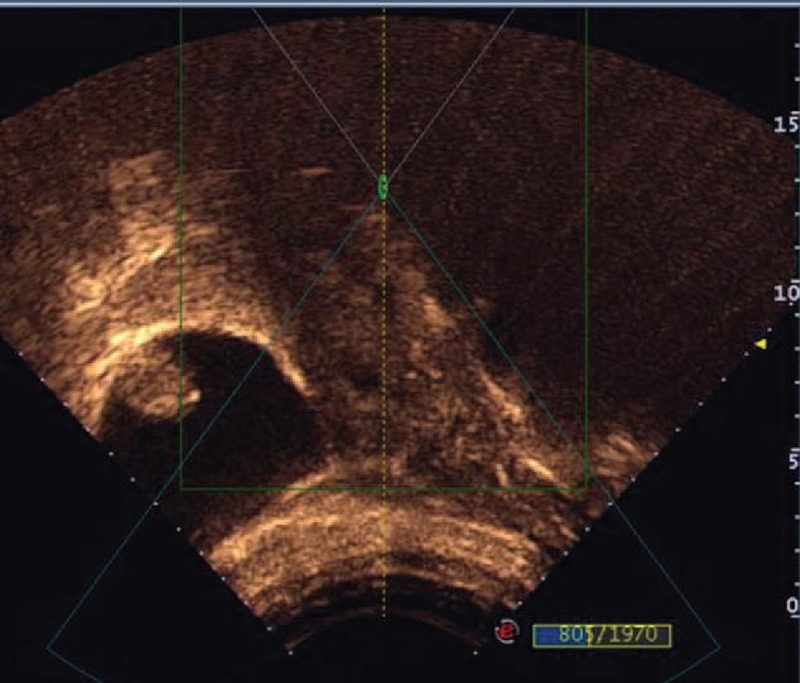
The blood flow of pregnancy tissue examined by transabdominal color Doppler scanning contrast medium revealed before high-intensity focused ultrasound ablation.

**FIGURE 6:**
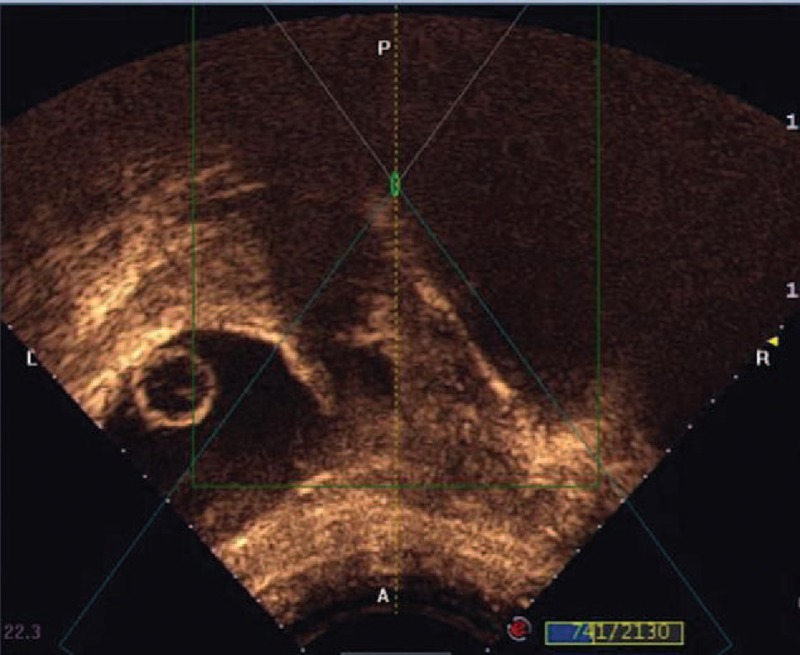
The blood flow of pregnancy tissue examined by transabdominal color Doppler scanning contrast medium after high-intensity focused ultrasound ablation.

All the patients received 1 session of HIFU ablation therapy. The median treatment time (defined as from the first sonication to the last sonication) was 73 (range: 13–160) minutes. The median HIFU sonication time was 600 (range: 100–1538) s. Before HIFU ablation, the color Doppler showed fetal cardiac activity in 20 cases. After HIFU treatment, the fetal cardiac activity disappeared. Contrast-enhanced ultrasound showed no perfusion in the pregnancy tissue immediately after the HIFU treatment (Figures 5 and 6).

As shown in Table 2, during HIFU treatment, 20 patients complained of sacral pain and 44 patients complained of lower abdominal pain. The pain score ranged from 1 to 2 points in 22 of 53 (41.5%) patients, and ranged from 3 to 4 points in 31 of 53 (58.5%) patients. Seven patients complained of a “hot” skin sensation, but no skin burn occurred. All the patients tolerated the treatment procedure well.

**TABLE 2:**
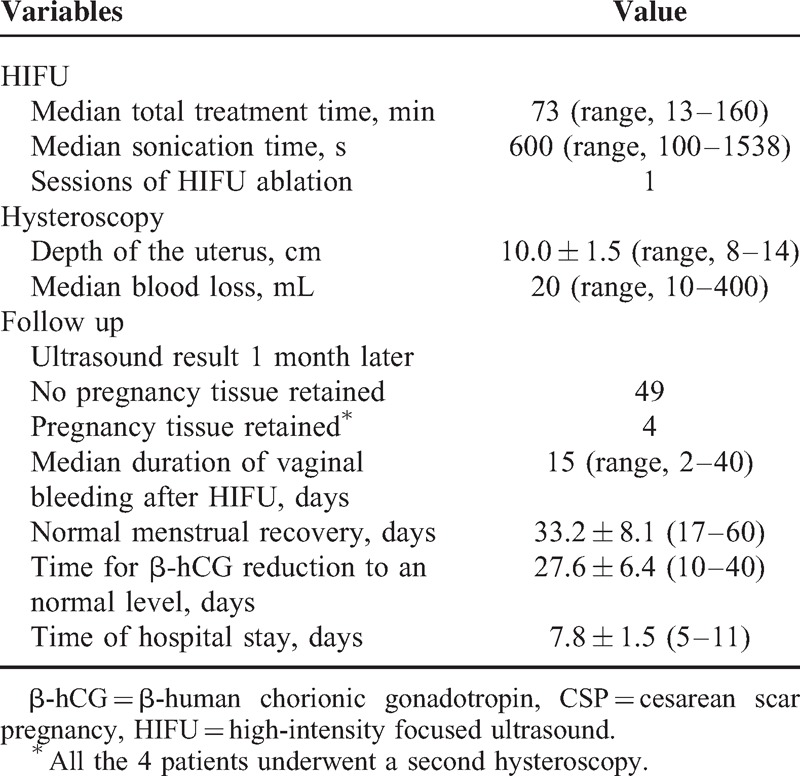
Treatment Results for Patients With CSP

### Evaluation of Suction Curettage under Hysteroscopic Guidance

All the patients received suction curettage under hysteroscopic guidance at 1 to 5 days after HIFU treatment. The average depth of the uteri was 10.0 ± 1.5 cm (range: 8–14 cm). The median volume of the intraoperative blood loss was 20 mL (range: 10–400 mL). Forty-nine patients underwent only 1 session of suction curettage under hysteroscopic guidance and all the pregnancy tissues were successfully removed. The remaining 4 patients underwent a second session of suction curettage under hysteroscopic guidance (Table 3). One of the 4 patients had a uterus perforation at the previous cesarean scar during the first suction curettage procedure and the procedure was terminated. During the follow-up period, the patient complained of a small volume of vaginal bleeding. She was requested to come back to take the second session of suction curettage under hysteroscopic guidance 1 month later. The procedure was successfully performed and the retained pregnancy tissue was removed. The other 3 patients mentioned retained pregnancy tissue at the 1-month follow-up point, and the second suction curettage under hysteroscopic guidance was performed. In 2 of the 3 patients, the CSP lesions were 52 and 60 mm in the larger diameters, and the volume of intraoperative blood loss was about 400 mL in the second suction curettage.

**TABLE 3:**
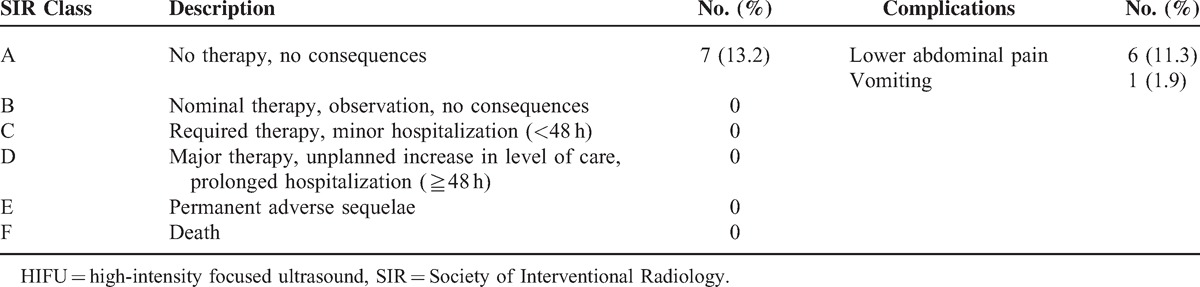
Incidence Rate of Adverse Effects or Complications After HIFU Treatment (n = 53)

### Follow-Up Result

The average hospital stay was 7.8 ± 1.5 (range: 5–11) days. All the patients complained of small vaginal bleeding after suction curettage. The median duration of vaginal bleeding was 15 (range: 2–40) days. The menstruation recovered within 2 months after HIFU treatment in 52 patients, whereas one patient recovered her menstruation using progesterone in 60 days after she was discharged from hospital. The average time of menstruation recovery was 33.2 ± 8.1 (range: 17–60) days. The time for the serum β-hCG level to return to normal was 27.6 ± 6.4 (range: 10–40) days. During the follow-up period, no catastrophic hemorrhage was observed and no further UAE or hysterectomy was performed (Table 2). No HIFU-related complications occurred.

## DISCUSSION

The major risks of CSP are uterine rupture and catastrophic hemorrhage. Therefore, the objectives of treatment for CSP are to eliminate the embryo, decrease the risk of bleeding, and preserve the uterus to maintain further fertility before the gestational sac rupture and hemorrhage.^4^ An individual treatment plan can be made based on the gestational age, the thickness of the intervening myometrium between the gestational sac and the bladder, the clinical symptom, the serum β-hCG level, and the desire for further fertility. In our institute, MTX treatment, UAE, hysteroscopic resection, laparoscopic resection, and HIFU are all available.

As a noninvasive treatment, HIFU has its advantage in the treatment of CSP. During HIFU treatment, the ultrasound beams from the transducer penetrated the abdominal wall and focused at the fetal tissue. The acoustic waves were converted to thermal energy, causing fetal tissue to heat up. When the temperature at the target increased to over 65°C, coagulative necrosis occurred.^19^ A previous study has also shown that HIFU can also destroy small vessels with a diameter <2 mm.^22^ Therefore, HIFU ablation could be used to kill CSP tissues and destroy small blood vessels around the CSP. Consequently, HIFU may help reduce the blood loss during the procedure of suction curettage under hysteroscopic guidance. Recently, Huang et al treated 4 CSP patients using HIFU combined with suction curettage. In their study, 2 patients were pretreated with MTX (50 mg, intramuscularly) for 8 days. Another patient was enrolled because the planned treatment of mifepristone followed by hysteroscopy had failed. HIFU treatment followed by successfully suction curettage was successful in these 3 patients. However, laparoscopic resection after suction curettage followed by HIFU treatment was performed in 1 patient with the largest gestational sac diameter of 52 mm because of persisting viginal bleeding. This patient did not receive pre-HIFU treatment with MTX and the gestational size was larger than that of the previous 3 cases.^19^ So, we asked whether CSP can be terminated by HIFU alone or whether the therapeutic results of HIFU are dependent on the size of the sac. In another study, Xiao et al treated 16 CSP patients with the average largest diameter of sac/mass at 24.8 ± 5.9 mm. Based on the size of the sac, the patients were treated in different sessions. After an average of 3.25 sessions of HIFU treatment, the β-hCG returned to normal level in all the patients and the sac/mass disappeared at different follow-up time points. Their results indicated that HIFU is effective for CSP patients at the early stage when the sac/mass is small. In the present study, our results showed that the median volume of blood loss of the 53 patients was 20 mL. The serum β-hCG returned to normal level at 27.6 ± 6.4 days after HIFU treatment, and the menstruation returned in all the 53 patients. Cao et al^23^ have treated 54 CSP patients with suction curettage after UAE. The average intraoperative blood loss was 25.0 ± 45.4 mL. In a comparison study, Gao et al retrospectively analyzed 119 CSP patients who were treated with UAE or systemic MTX followed by dilation and curettage. Their results showed that blood loss was 261.0 ± 357.4 mL in the MTX group, whereas it was 14.1 ± 40.6 mL in the UAE group; the serum β-hCG returned to normal level at 40.5 ± 17.2 days in the MTX group, whereas in the UAE group, it returned to the normal level at 15.4 ± 7.7 days.^24^ Our results demonstrated that HIFU followed by suction curettage is effective in treating CSP and it seems to be superior to MTX followed by suction curettage, and similar to UAE in combination with suction curettage.

The safety of this non-invasive technique in the treatment of CSP patients is always a concern. In this study, immediately after HIFU treatment, we observed the common adverse effects of lower abdominal pain, and vomiting. The rate of adverse effects of HIFU followed by suction curettage is less than that of MTX or UAE followed by suction curettage.^7–9^ These adverse effects can be explained by uterus contraction after HIFU. Based on the Society of Interventional Radiology classification system for complications by outcome, these adverse effects were mild and most of them subsided within 1 day without any therapy.^25^ Although these patients had abdominal surgical scars and some of them reported temporary “hot” skin sensations during the HIFU procedure, no skin burn occurred. The other HIFU-related adverse effects included nerve injury and bowel injury, which both did not occur in this study. Although we observed 2 cases with intraoperative blood loss around 400 mL during suction curettage, the incidence is just similar to that of UAE followed by suction curettage. We further reviewed these 2 patients and found that the CSP lesions were 52 and 60 mm in the largest diameter, but the sonication time was about the same as we used to treat the patients with smaller CSP lesions. We may decrease this adverse effect by increasing the sonication time to deposit more energy to heat the target tissue. In this study, uterine perforation at previous cesarean scars occurred during the hysterscopic procedure in the first session of HIFU treatment. We terminated the procedure and successfully performed the second session of suction curettage under hysteroscopic guidance. We further reviewed this case and found that the thickness of the intervening myometrium between the gestational sac and the bladder was only 2 mm. To treat patients with thin thickness of the intervening myometrium between the gestational sac and the bladder, the procedure of suction curettage under hysteroscopic guidance should be performed carefully.

This study was limited because it was a retrospective study and we did not compare HIFU ablation followed by suction curettage with MTX treatment or UAE followed by suction curettage. This study was also limited by the relatively small number of patients and the lack of multiple centers’ data. Another limitation of this study was that we only enrolled patients with gestation ages <8 weeks. Future studies to enroll patients with gestational age >8 weeks are needed. Although a previous study has shown that it is not necessary to perform suction curettage to remove the CSP tissue after HIFU ablation, the sample size was too small. Therefore, future studies with larger sample sizes than this study to compare HIFU followed by suction curettage with HIFU without suction curettage are needed. Although HIFU could be used safely to terminate CSP, it still has the risk of massive vaginal bleeding during the suction curettage and hysterscopic procedure. Many studies have shown that MTX could kill CSP fetal tissue. Thus, injection of MTX before HIFU ablation may decrease the activity of the chorionic villi, and the thermal energy might be easier to deposit in the CSP lesion, which resulted in easier necrosis of CSP and destroyed small vessels around CSP. Future studies combining MTX with HIFU are also needed.

## CONCLUSION

Based on our results with a relatively small number of patients, it appears that USgHIFU combined with suction curettage under hysteroscopic guidance is a safe and effective modality to treat CSP patients when the gestational period is >8 weeks. Future multicenter studies to compare USgHIFU followed by suction curettage with MTX or UAE and then with suction curettage, or MTX combined with HIFU followed by suction curettage under hysteroscopic guidance are needed.
